# Anti‐TNF Drug‐Induced Sarcoidosis in Inflammatory Bowel Diseases: Multicentric Case Series and Literature Review

**DOI:** 10.1155/bmri/7554982

**Published:** 2026-05-22

**Authors:** Patrick Bez, Zlata Chkolnaia, Annalisa Aratari, Marta Ascolani, Péter Bacsur, Andreas Blesl, Philippe Seksik, Sara Nikolic, Savarino Edoardo V., Marcello Rattazzi, Francesco Cinetto, Carla Felice

**Affiliations:** ^1^ Rare Diseases Referral Center, Internal Medicine 1, Ca′ Foncello Hospital, AULSS2 Marca Trevigiana, Treviso, Italy; ^2^ Gastroenterology Department, Sorbonne Université, INSERM, Centre de Recherche Saint-Antoine, CRSA, AP-HP Saint-Antoine Hospital, Paris, France, sorbonne-universites.fr; ^3^ IBD UNIT, San Filippo Neri Hospital, Rome, Italy, sanfilipponeri.roma.it; ^4^ Gastroenterology Unit, Ca′ Foncello University Hospital, Treviso, Italy; ^5^ Center for Gastroenterology, Albert Szent-Györgyi Medical School, University of Szeged, Szeged, Hungary, u-szeged.hu; ^6^ Division of Gastroenterology and Hepatology, Department of Internal Medicine, Medical University of Graz, Graz, Austria, medunigraz.at; ^7^ Department of Gastroenterology, University Medical Center Maribor, Maribor, Slovenia, ukc-mb.si; ^8^ Department of Surgery, Oncology and Gastroenterology, University of Padova, Padova, Italy, unipd.it; ^9^ Department of Medicine (DIMED), University of Padova, Padova, Italy, unipd.it

**Keywords:** anti-TNF, drug-induced sarcoidosis, inflammatory bowel disease, infliximab, sarcoidosis

## Abstract

**Introduction:**

Sarcoidosis is an inflammatory granulomatous condition and presents overlapping features with inflammatory bowel disease (IBD). Anti‐TNF treatment has revolutionized the management of several conditions, including IBD and sarcoidosis. Yet, anti‐TNF drugs have been associated with drug‐induced sarcoidosis reaction (DISR).

**Methods:**

This is a retrospective, international, multicentric case series, including IBD patients with anti‐TNF–related DISR. A literature review was performed to identify previously published cases.

**Results:**

Nine new cases of anti‐TNF–related DISR in IBD are described with a long follow‐up (median 45 months). After literature review, a total of 26 cases were identified. The diagnosis required histological evidence of granulomas in involved organs in all patients. Most patients had a diagnosis of Crohn′s disease (*n* = 19, 73.1%). The culprit drug was infliximab in 15 (57.7%) and adalimumab in 11 (42.3%). The median time to sarcoidosis development was 21 months. Compared to conventional sarcoidosis, extrathoracic involvement was more prevalent (*n* = 20, 76.7%). Management of DISR consisted of discontinuing anti‐TNF treatment in 20 cases (76.9%) and providing specific treatment in 17 cases (65.4%), with favorable outcomes in all cases.

**Conclusion:**

Despite its rarity, DISR may be challenging for IBD patients. Discontinuation of anti‐TNF treatment is recommended, and specific treatment is required for moderate‐to‐severe cases.

## 1. Introduction

Inflammatory bowel diseases (IBD) encompass a spectrum of conditions including two major entities, ulcerative colitis (UC) and Crohn′s disease (CD), that may lead to progressive gastrointestinal tissue damage and disability [1, 2] and are associated with a high burden of disease [3]. The main therapeutic targets are symptom resolution, mucosal healing, a normal quality of life, and the prevention of complications [2]. Several medical therapies are currently approved for the treatment of IBD, including mesalamine, steroids, immunomodulators (such as azathioprine), and advanced therapies, including anti‐tumor necrosis factor‐alpha (anti‐TNF) drugs, newer biological drugs, and JAK (Janus‐kinase) inhibitors [4]. These treatments have contributed to reducing the 5‐year surgery rate for CD and the rate of colectomy for UC [3].

Sarcoidosis is a rare idiopathic multiorgan granulomatous disease that can affect any organ [5]. The pathogenesis of this condition is complex, involving alterations to the innate and adaptive immune system [6] and environmental exposure [7]. Intrathoracic involvement, affecting the hilar–mediastinal lymph nodes and/or the lungs, occurs in 90% of cases. Extrapulmonary involvement may potentially affect any organ and occurs in up to 50% of cases. Skin and eye involvement are considered more common, but rare organ involvement, such as heart and central nervous system, can be life‐threatening [5]. Interestingly, sarcoidosis and IBD share possible multisystemic organ involvement and genetic loci predisposition [8, 9]. IBD primarily affects the gastrointestinal tract, whereas sarcoidosis generally affects the lung, the skin, or the eyes, suggesting that environmental factors and microbiome are involved in their pathogenesis [7]. In addition, CD and sarcoidosis are both granulomatous conditions [10].

Anti‐TNF drugs are currently considered first‐line agents for IBD in most countries, due to their efficacy and relatively low cost following the introduction of biosimilars [4]. The same class of drugs is approved for treating other immune conditions, including psoriasis and various rheumatological disorders, and is also used to treat several rare inflammatory diseases, such as sarcoidosis [11]. During postmarketing surveillance, anti‐TNF drugs have been associated with several paradoxical reactions, including psoriasis [12], drug‐induced sarcoidosis reaction (DISR) [13], and even IBD [14]. Although paradoxical psoriasis in IBD has been extensively characterized [15], anti‐TNF DISR in IBD is rare and has only been reported in individual case reports and/or small case series [13, 16–30]. Here, we present new cases of IBD patients with anti‐TNF DISR and review the currently available literature to better characterize this rare but challenging clinical condition.

## 2. Materials and Methods

This is a retrospective case series including patients from several countries across Europe. Data were obtained from medical records, registered in an anonymous shared database in June 2025, and included demographics, IBD characteristics (diagnosis, duration, comorbidities, prior medications, indication to anti‐TNF treatment, disease activity, and treatment regimen at the time of sarcoidosis diagnosis), and sarcoidosis features (organ involvement, diagnostic tests, histopathology, symptoms, and therapy). Also, clinical outcomes and follow‐up after sarcoidosis diagnosis were recorded. Written informed consent for data retrieval from medical records was obtained from patients. All anonymized cases were discussed with sarcoidosis experts (P.B. and F.C.) at the tertiary referral Center for Rare Immunological Disorders in Treviso (Italy).

For the literature review, we searched PubMed for English language articles from January 1, 2001, to June 30, 2025, excluding animal studies. As we are dealing with rare cases, we did not exclude reviews, as this would possibly mean missing relevant case reports. The following terms were used: “paradoxical sarcoidosis,” “anti‐TNF drug‐induced reaction,” “sarcoidosis and Crohn′s disease,” “sarcoidosis and ulcerative colitis,” and “sarcoidosis and inflammatory bowel disease.” We selected only reports on cases with a diagnosis of anti‐TNF DISR for IBD.

After removing duplicates, we obtained 612 unique articles. After screening the articles by reading the abstracts, we identified 13 articles with 14 unique case reports. By reading the full texts of these articles, as well as articles used for preparing the manuscript, we identified three additional relevant case reports. We searched for the clinical information mentioned above. Where relevant data on specific cases were missing, we directly contacted the corresponding author to obtain additional information

Categorical variables were summarized with absolute and relative frequencies, whereas the continuous variables were summarized using the median and interquartile range (IQR).

## 3. Results

### 3.1. Original Case Series

Our cohort included nine patients with sarcoidosis occurred during treatment with anti‐TNF agents for IBD: six CD (66.7%), two UC (22.2%), and one IBD unclassified (IBD‐U, 11.1%). The median age at IBD diagnosis and at start of anti‐TNF treatment was 22 years (IQR 20–30) and 34 years (IQR 25–51), respectively. Most patients were male (8, 88.9%); the only female patient had UC. Six patients (66.7%) presented with extraintestinal manifestations (EIMs): In particular, four had spondyloarthritis, two had cutaneous, and one had hepatic involvement (one patient presented both joint and skin disease). Tables 1 and 2 summarize the clinical characteristics of the entire cohort and of the individual patients, respectively. Table S1 describes IBD characteristics according to the Montreal classification [31] and EIM. The CD patients often presented ileocolonic involvement (L3: 4/6), gastric involvement (L4: 4/6), and perianal disease (p: 3/6). The disease behavior was penetrative (B3) in 3/6 patients and structuring (B2) in 2/6, with a high rate of previous surgery (5/6), suggesting a high disease burden. The two UC patients presented with left‐sided colitis (E2) and extensive colonic involvement (E3), respectively. The IBD‐U patient presented with extensive colonic involvement.

**Table 1:** Summary of the clinical characteristics of the cohort.

Variable	Whole cohort (*N* = 26)	Original series (*N* = 9)	Literature review (*N* = 17)
IBD (CD/UC/IBD‐U)	19 (73.1)	6 (66.7)	13 (76.5)
	6 (23.1)	2 (22.2)	4 (23.5)
	1 (3.85)	1 (11.1)	0 (0)
Sex (M/F)	17 (65.4)	8 (88.9)	9 (52.9)
	9 (34.6)	1 (11.1)	8 (47.1)
Anti‐TNF drug (IFX/ADA)	15 (57.7)	5 (55.6)	10 (58.8)
	11 (42.3)	4 (44.4)	7 (41.2)
Age at onset of IBD	22 (19–32)	22 (20–30)	22 (20–31)
Age at anti‐TNF treatment start (y)	35 (28–44)	34 (25–51)	35 (30–42)
Time interval from initiation of anti‐TNF drug to sarcoidosis development (months)	21 (12–60)	14 (8–60)	24 (12–60)
Sarcoidosis specific symptoms	16 (61.5)	6 (66.7)	10 (58.8)
Histology (available)	26 (100)	9 (100)	17 (100)
Sarcoidosis involvement			
Intrathoracic involvement	22 (84.6)	8 (88.9)	14 (82.4)
Extrathoracic involvement	20 (76.9)	6 (66.7)	14 (82.4)
Skin	9 (34.6)	3 (33.3)	6 (35.3)
Subdiaphragmatic LN	4 (15.4)	2 (22.2)	2 (11.8)
Liver	3 (11.5)	0 (0)	3 (17.6)
Spleen	3 (11.5)	1 (11.1)	2 (11.8)
Kidney	2 (7.69)	1 (11.1)	1 (5.88)
Central nervous system	2 (7.69)	1 (11.1)	1 (5.88)
Eye	1 (3.85)	0 (0)	1 (5.88)
Heart	1 (3.85)	0 (0)	1 (5.88)
Other organs		1 (11.1), Salivary glands (*n* = 1)	2 (11.8), Gut (*n* = 1), oral mucosa (*n* = 1)
Sarcoidosis treatment			
Anti‐TNF drug interruption	20 (76.9)	9 (100)	11 (64.7)^a^
Specific treatment	17 (65.4)	5 (55.6)	12 (70.6)
Steroid	17 (65.4)	5 (55.6)	12 (70.6)
Hydroxychloroquine	1 (3.85)	0 (0)	1 (5.88)
Azathioprine	1 (3.85)	1 (11.1)	0 (0)
Follow‐up (months)	14 (3–35)	45 (22–52)	7.5 (3–15)
Progression and death	1 (3.85)	1 (11.1)	0 (0)

*Note:* The whole cohort encompasses both the new patients described in the original series and the already described patients from the literature review. The categorical variables are represented as absolute numbers and percentages, whereas continuous variables are represented as median and interquartile range (IQR).

Abbreviations: ADA, adalimumab; F, female; IBD, inflammatory bowel disease; IBD‐U, unclassified IBD; IFX, infliximab; LN, lymph node; M, male; TNF, tumor necrosis factor; UC, ulcerative colitis; y, year.

^a^One patient started again infliximab.

**Table 2:** Characteristics of the new nine patients with anti‐TNF–induced sarcoidosis and IBD.

ID	Sex	IBD	Montreal	EIM	Anti‐TNF drug	Time to dx (y)	SS	Organ involvement	Organ biopsied	BAL (CD4/CD8 ratio)	Anti‐TNF stop	Therapy	FU (m)/response	New therapy for IBD
MUG‐01	M	CD	A2 L3 + L4 B1p	A	ADA	7.5	No	Neuro, lung, skin	Lung	24.7	Yes	Steroid	21/CR	USTE
PSAT‐01	M	CD	A2 L3 + L4 B3p	A + S	IFX	1.1	Yes	Lung	Lung	NA	Yes	Steroid	52/PR	GUSE
PSAT‐03	M	CD	A2 L1 B3		ADA	0.67	Yes	Lung, skin, kidney, salivary glands, heart (?)	Lung, kidney, salivary glands	NA	Yes	Steroid AZA	45/CR	USTE
SZE‐01^a^	M	CD	A2 L2 B2	S	IFX	1.2	No	Lung, skin	Lung	NA	Yes	Steroid	125/R, relapse	ADA (with relapse), then USTE
PD‐01	M	CD	A3 L3 + L4 B3p		ADA	5	Yes	Lung, uLN, lLN	Lung	5.0	Yes	No	22/CR	USTE
TV‐01	M	CD	A1 L3 + L4 B2p		IFX	7.6	No	Lung, lLN	Lung	20	Yes	Steroid	14/PR	UPA
MB‐01	F	UC	E2	A	ADA (before IFX)	0.2	No	Skin	Skin	No	Yes	No	130/CR	VEDO
TV‐02	M	UC	E3	H	IFX	0.4	No	Lung	Lung	0,62	Yes	No	28/PR	VEDO
PSAT‐02	M	IBD‐U	E3	A	IFX	1.3	Yes	Lung	Lung	Not known	Yes	No	47/CR	VEDO

Abbreviations: A, articular; ADA, adalimumab; BAL, bronchoalveolar lavage; CD, Crohn′s disease; dx, diagnosis of IBD; EIM, extraintestinal manifestation; F, female; GUSE, Guselkumab; H, hepatic; IBD, inflammatory bowel disease; IBD‐U, undefined IBD; IFX, infliximab; lLN, infradiaphragmatic lymph nodes; Lung, lung involvement, including hilar–mediastinal lymph nodes; M, male; Montreal, classification according to Montreal for UC and/or CD [31]; Neuro, neurological involvement; S, skin; SS, systemic symptoms for sarcoidosis; UC, ulcerative colitis; uLN, sovradiaphragmatic lymph nodes excluding hilar–mediastinal lymph nodes; UPA, upadacitinib; USTE, ustekinumab; VEDO, vedolizumab; y, years.

^a^Deceased for unrelated complication.

The most frequent indication (in some cases more than one) for anti‐TNF treatment was no response/intolerance to conventional therapies (*n* = 5). Other indications included: perianal disease (*n* = 2), steroid refractoriness (*n* = 1), steroid dependence (*n* = 1), prevention of postoperative recurrence (*n* = 1), and EIM (*n* = 1). The culprit drug was infliximab in five cases (55.6%) and adalimumab in four (44.4%).

At the time of sarcoidosis diagnosis, all patients were in clinical remission/response under anti‐TNF treatment; in four cases, mucosal healing was also described. The infliximab dose was optimized in 3/5 patients at the time of sarcoidosis diagnosis.

The median time from the start of anti‐TNF treatment to the development of sarcoidosis was 14 months (IQR 8–60). In 6/9 (66.6%) cases, sarcoidosis presented with symptoms, such as fever (*n* = 5), cough (*n* = 3), arthralgia (*n* = 2), and weight loss (*n* = 2), and in one case neurological symptoms (see below the case #MUG1). The most frequent localization was intrathoracic (*n* = 8) with enlarged mediastinal lymph nodes in eight cases and lung micronodules in six cases. Extrathoracic involvement was frequent (*n* = 6, 66.7%). The commonly affected organs were the skin (*n* = 3, one piercing and tattoo involvement, one erythema nodosum, and one not specified) and the abdominal lymph nodes (*n* = 2). One CD patient (#PSAT‐003, Table 2) presented a multiorgan disease with lung, skin, kidney (interstitial nephritis), and salivary glands involvement. He also developed angina, but coronarography and cardiac ultrasound were unremarkable and cardiac magnetic resonance imaging (MRI) and/or cardiac positron‐emission computer tomography (PET‐CT) were not performed; therefore, it is not possible to definitively exclude cardiac sarcoidosis. Another CD patient (#MUG‐1) presented with vertigo, visual disturbance, and neck pain: the cerebrospinal fluid examination excluded infections and confirmed the diagnosis of aseptic meningitis with papilledema, with subsequent PET‐CT showing mediastinal enlarged lymph nodes that were biopsied to confirm diagnosis of sarcoidosis (probable neurosarcoidosis according to Neurosarcoidosis Consortium Consensus Group [32]).

Bronchoalveolar lavage (BAL) supported the diagnosis of sarcoidosis in six cases with lung involvement. The CD4+/CD8+ T‐cell ratio in BAL fluid was available in four cases with a diagnostic result (i.e., a ratio greater than 3.5) in 3/4 cases (25, 20, 5, and 0.62, respectively). Histological confirmation of sarcoidosis showing the presence of noncaseating granuloma was reported in all patients. The biopsied organs included lung parenchyma or mediastinal lymph nodes in seven patients, as well as skin, minor salivary glands, and kidney in single cases. Notably, one patient (#PSAT‐03) was biopsied in multiple organs (Table 2).

The median peripheral lymphocyte count at diagnosis was 1400 cell/mm^3^ (IQR 1200–1700). Increased soluble angiotensin converting enzyme (sACE) and hypergammaglobulinemia at diagnosis were reported in 4/6 and 2/4 patients with available data. For one CD patient (#TV‐001), soluble interleukin‐2 receptor (sIL‐2R) and interleukin‐6 (IL‐6) were available and were both increased.

The treatment of sarcoidosis consisted in anti‐TNF drug discontinuation for all patients, steroid administration in five patients, and add‐on therapy with azathioprine in one case with multiple organ involvement including renal disease (#PSAT‐03). Later, alternative advanced treatments for IBD were started, including vedolizumab (*n* = 3), ustekinumab (*n* = 3), upadacitinib (*n* = 1), and guselkumab (*n* = 1). In one case (#SZE‐001), after induction of remission of sarcoidosis through steroids and discontinuation of anti‐TNF treatment (infliximab), the patient suffered a flare of CD requiring surgery; thus, adalimumab was introduced as postoperative prevention therapy, during which sarcoidosis relapsed. The patient was then treated again with high‐dose steroids with definitive resolution of sarcoidosis. After 2 years of adalimumab monotherapy, the patient required diverting transverse ostomy for worsening of perianal disease. Finally, he was switched to ustekinumab and did not experience new relapses of sarcoidosis. The patient, a heavy smoker, died 4 years later from unrelated complications of chronic obstructive pulmonary disease at the age of 52.

A further patient with UC was reported as having developed sarcoidosis, but 8 years after anti‐TNF drug withdrawal and therefore excluded from this case series because a causal link between biologic treatment and sarcoidosis onset cannot be confirmed.

### 3.2. Cases From Literature Review

We found 16 articles reporting 17 cases of anti‐TNF DISR in IBDs. Fifteen cases were reported in form of case reports [16–22, 24–30], whereas two cases were reported in a case series and literature review on anti‐TNF DISR, including rheumatological patients [13]. Table 1 summarizes the characteristics of all patients in the cohort, whereas Table S2 provides a detailed description of individual cases from the literature. Of the patients suffering from anti‐TNF DISR, 13 cases (76.4%) had CD and four had UC (23.5%), with no sex prevalence (52.9% male). The culprit drugs were infliximab in 10 cases (58.8%) and adalimumab in seven (41.2%). Lung involvement occurred in most patients (*n* = 14, 82.3%), presenting as hilar–mediastinal adenopathy and/or lung parenchyma involvement. Extrathoracic involvement was also very common (*n* = 14, 82.3%). Common extrathoracic complications included cutaneous involvement (*n* = 6) and hepatic involvement (*n* = 2). Single case reports described multisystemic sarcoidosis involving the eyes (uveitis) [13], heart [13], spleen [24], and a combination of the kidneys and gut [18]. In addition, patients with isolated oral involvement [26] and isolated neurological involvement were reported. In the latter case, sarcoidosis diagnosis was confirmed by a spinal biopsy [16] (definite neurosarcoidosis according to Neurosarcoidosis Consortium Consensus Group [32]).

Regarding the laboratory markers in support of sarcoidosis diagnosis, the reported data were very scarce and heterogeneous: sACE was elevated in 4/9 cases studied (44.4%), C‐reactive protein was elevated in 2/3 cases, and sIL‐2R was elevated in 1/2 cases. In a single report [29], lysozyme and TNF levels were elevated. BAL results were described in five patients, with the CD4/CD8 ratio above 3.5 (diagnostic for sarcoidosis) in 3/5 patients tested. Histological findings were available in all cases.

At time of DISR detection, IBD was generally under good control with anti‐TNF therapy, except in one case that occurred during a disease flare of IBD requiring treatment for both conditions [13]. The treatment of sarcoidosis consisted in anti‐TNF drug discontinuation in 11 cases (64.7%) and introduction of specific therapy for sarcoidosis in 12 cases (70.6%). The first‐line agent was a steroid in 10 cases, a topical steroid in one case [26], and hydroxychloroquine with topical steroids in another case [13]. In the latter case, due to disease progression of DISR with lung function deterioration and detection of cardiac involvement, high‐dose steroids were started with improvement of lung function and cutaneous involvement in 1 month.

In detail, anti‐TNF therapy discontinuation was the only intervention for three mild cases [19, 28, 29], whereas eight more patients required the addition of a specific treatment [13, 16–18, 21, 23, 27]. In general, these interventions led to the resolution (or initial response) of sarcoidosis within the available follow‐up. In one of the mild cases, the patient was rechallenged with infliximab and did not experience a relapse of sarcoidosis during the subsequent 19 months of follow‐up [19]. Notably, in one case with a long follow‐up, ustekinumab was introduced as an alternative treatment for IBD after sarcoidosis remission was induced, with no relapse occurring during the subsequent 6 months of follow‐up [27]. Anti‐TNF drug was continued in the remaining six patients. These cases were generally mild, with most (4/6) being published before 2016. In two patients [20, 25], the drug was carefully continued without additional intervention. In the long‐term follow‐up period (6–9 months), lung manifestations improved spontaneously, whereas skin manifestations resolved in only one case. Specific treatment for sarcoidosis resulted in resolution (or an initial response) of the disease in two of the four remaining cases. In one case, each infusion of infliximab was premedicated with a high dose of steroids (with a 3‐year follow‐up period). In the last case, who presented with isolated oral involvement, the DISR presented with relapse‐remitting flares that required courses of topical steroid. None of the 17 patients presented with a progressive disease course or died during the follow‐up.

## 4. Discussion

So far, a total of 26 IBD patients with anti‐TNF–induced sarcoidosis have been described, with nine additional cases being added to those already documented in the literature. Despite several new advanced therapies currently being approved for the treatment of IBD, anti‐TNF treatment remains the principal and usually the first biological approach, especially after the introduction of biosimilars [4]. Anti‐TNF DISR is a very rare, yet highly impacting, paradoxical manifestation that requires treatment interruption for IBD and switch to alternative more expensive therapies, often requiring steroid courses.

A definitive sarcoidosis diagnosis requires evidence of granulomas in a biopsy sample, along with the exclusion of other possible causes of granulomatous diseases (especially mycobacterial infections) [33]. In cases of paradoxical sarcoidosis, a temporal association between drug initiation and symptom onset is required [34]. In our series, all patients presented with histological evidence of granulomas in various organs and infectious diseases were reasonably ruled out through noninvasive ancillary tests (e.g., sputum) and/or invasive tests (BAL and liquor puncture) and histological techniques. Nonnecrotizing granulomas are a hallmark of both sarcoidosis and CD, despite being identified in nearly 20% of patients [1]. Histologically, a granuloma is defined as a “compact (organized) collection of mature mononuclear phagocytes, which is not necessarily accompanied by accessory features such as necrosis” [35]. Macrophage aggregation is considered to be caused by a persistent antigen. The pattern of inflammation can be influenced by the cytokine milieu with interferon‐gamma–driven M1‐polarization in the initial phases of sarcoidosis and inflammatory CD, which can turn into interleukin‐4/13–driven M2‐polarization in the late phases of sarcoidosis and stenosing CD, characterized by fibrosis [10]. Furthermore, sarcoidosis and CD share the common susceptibility locus 10p12.2 [9], and NOD2 mutations or polymorphisms have been described in early‐onset sarcoidosis and CD, respectively [8]. A recent GWAS study of European ancestry showed that genetic predisposition to IBD (both CD and UC) increases the risk of sarcoidosis, but genetic predisposition to sarcoidosis increases only the risk of CD [36]. Not surprisingly, several reports have described sarcoidosis in CD [37], but also, albeit more rarely, in UC [38] without anti‐TNF exposure. IBD is usually diagnosed before sarcoidosis, but concomitant or later diagnosis have been reported [39]. In addition, lung involvement in IBD is considered a rare (< 1%) EIM, with different types of manifestation, including necrobiotic pulmonary nodules [40]. Despite this, small observational studies have shown that a significant proportion of IBD patients exhibit alterations in pulmonary function tests (PFTs), suggesting that subclinical lung involvement may be an underestimated problem [41, 42]. As PFTs and lung imaging are not routinely performed in IBD, we cannot definitively confirm whether anti‐TNF treatment caused the granuloma or whether it may have exacerbated an unrecognized pre‐existing condition. However, in our series, sarcoidosis mainly occurred in patients with a partial or complete clinical response to anti‐TNF treatment, suggesting that DISR and IBDs are indeed separate clinical entities. We can speculate that the similarities between CD and sarcoidosis may explain why DISRs were more frequently observed in CD than UC in our series and in the literature.

When we compared the patients in our original series with those in the literature review, we found that the frequencies of IBD diagnosis, age at IBD onset, and age at anti‐TNF initiation were similar. This suggests that the two subcohorts can be compared. However, the time interval from anti‐TNF drug initiation to sarcoidosis tended to be shorter in our original case series than in the literature review (median 14 vs. 24, respectively). The frequency of intrathoracic was similar between the two subcohorts (88% vs. 82%), whereas extrathoracic involvement was slightly more frequent in the cases reported by the literature (67% vs. 82%).

**Figure 1:**
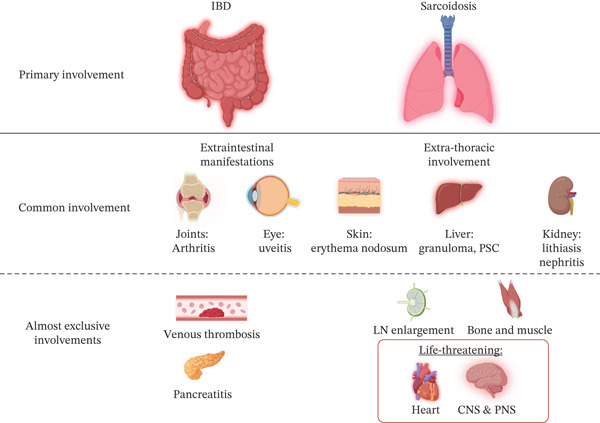
Comparison of principal organ involvements between inflammatory bowel diseases (IBD) and sarcoidosis [5, 44, 47]. CNS, central nervous system; IBD, inflammatory bowel disease; LN, lymph nodes; PNS, peripheral nervous system; PSC, primary sclerosing cholangitis. This figure was created using BioRender.

Considering the whole cohort, we found that intrathoracic involvement affected 22/26 cases (85%), which is similar to what is reported for sarcoidosis not related to anti‐TNF treatment (90%) [5]. The most common manifestations were hilar–mediastinal lymphadenopathy and/or lung involvement with micronodules. No cases of lung fibrosis were observed. Extrathoracic involvement was reported more frequently in our study (*n* = 20, 77%) than in conventional sarcoidosis (up to 50%) [5]. Sarcoidosis can affect the skin, eye, liver, and other organs. The involvement of the kidney, heart, or central nervous system is considered life‐threatening [5]. In our series and literature review, the skin was frequently affected with nonspecific manifestations (i.e., erythema nodosum) and specific manifestations, including scar sarcoidosis [43]. Interestingly, erythema nodosum, uveitis, and dactylitis are considered possible manifestations of both sarcoidosis and IBD [1, 2]. EIMs occur in 24% of UC and 35% of CD according to a recent meta‐analysis [44]; conversely, sarcoidosis may involve the gastrointestinal tract in 0.1%–3.4% of cases [45], including the appendix [46]. Once again, these data support that IBDs and sarcoidosis may be more closely related. Figure 1 and Table S3 compare the possible organ involvement between IBD and sarcoidosis.

Due to the variety of organs involved by sarcoidosis, the diagnosis may be challenging, with samples taken from several organs, including hilar–mediastinal lymph nodes, skin, kidney, intestine (in one UC patient), salivary glands, oral mucosa, and even—in one case—the spinal cord. The latter furnished the definitive proof of isolated neurosarcoidosis [16]. In another case (#MUG‐1), the evidence of granuloma in another organ (i.e., hilar–mediastinal lymph nodes) supported the diagnosis of neurosarcoidosis (probable neurosarcoidosis according to the Neurosarcoidosis Consortium Consensus Group [32]).

Several biomarkers that aid the diagnosis of sarcoidosis have been proposed, including sACE, sIL‐2R, CRP, and lysozyme [48]. sACE was elevated in 8/15 (53%) in our series, similar to what is reported for sarcoidosis [48]. The marker presents low sensitivity but relatively high specificity. Data on other biomarkers are scarce and therefore unreliable. Additional elements that support sarcoidosis diagnosis include elevation of CD4/CD8 ratio in BAL above 3.5 (which is considered diagnostic in specific settings), which was identified in 6/9 patients (67%) [5]. However, low levels of CD4/CD8 have been reported in up to half of patients with sarcoidosis, and this was associated with a more severe disease course [49].

In their narrative review, Andolfi et al. identified 111 patients affected by anti‐TNF–induced paradoxical sarcoidosis in various conditions, including patients with IBD treated with adalimumab and infliximab [34]. They confirm the previous observation by Decock et al. [13] that the most frequent drugs associated with sarcoidosis were etanercept (*n* = 57, 51%; in two cases with concomitant treatment with adalimumab and in one case with infliximab), adalimumab (*n* = 30, 27%), and infliximab (*n* = 21, 19%). Certolizumab and golimumab were reported in one clinical case each [34]. In our series, the drugs most frequently associated with paradoxical sarcoidosis in IBD were infliximab (*n* = 15, 58%) and adalimumab (*n* = 11, 42%). We report no cases under certolizumab (approved for CD) or golimumab (approved for UC) [4]. Etanercept is not approved for IBD. The time from the beginning of anti‐TNF therapy and the diagnosis of sarcoidosis varies widely with a median of 21 months and a range from a few months up to several years of treatment. This is similar to what is reported in the current literature [34].

Far from being a specific complication of anti‐TNF drugs, DISRs have been described during treatment with several agents including immune checkpoint inhibitors (ICIs), BRAF inhibitors, several monoclonal antibodies (natalizumab, tocilizumab, rituximab, ustekinumab, trastuzumab, and dupilumab), and also interleukin inhibitors, such as abatacept [34]. This suggests that paradoxical reactions can occur through different immune‐mediated mechanisms, extensively reviewed elsewhere [6]. The exact pathophysiologic mechanism that leads to the development of DISR during anti‐TNF therapy is not completely understood. Immunogenicity (i.e., the formation of anti‐drug antibodies, ADAs) represents a risk for the occurrence of secondary failure and adverse events, especially with infliximab [50]. In our series, we do not have data on drug trough levels and ADA; however, given that most patients were in sustained remission at the time of DISR diagnosis, we can speculate that immunogenicity is not involved in its pathogenesis.

As for other paradoxical drug reactions, the first therapeutic approach is the discontinuation of the offending drug and, only in more severe cases, the addition of steroids or immune‐suppressive drugs. The decision to stop the drug should be guided by the following considerations: (1) the severity of DISR, (2) the availability of alternative treatments for IBD, and (3) the severity of the primary conditions [34] In the nine original patients described, anti‐TNF drug was discontinued in all cases, whereas in some cases from the literature review, it was continued (*n* = 6) or suspended only temporarily (*n* = 1). These cases were generally mild, and most were published before 2016, when anti‐TNF drugs were the only biologics available; therefore, their discontinuation could result in surgery. In most of these cases, only partial resolution of sarcoidosis was observed, with some having relapse‐remitting behavior. In our series, a specific therapy for sarcoidosis was required in 18/26 (69%) and systemic steroid resulted in an initial or complete response in most cases during the available follow‐up. Azathioprine was required as add‐on therapy in one case characterized by granulomatous interstitial nephritis. Despite the same anti‐TNF medication being safely restarted in one mild case (#14) [20] and continued in six patients in the literature review, one of our patients (#SZE‐001) experienced a sarcoidosis relapse after a different anti‐TNF was introduced. Thanks to the long follow‐up in our case series (median 45 months compared to 14 months in the literature series), we can conclude that patients do not develop long‐term lung complications from DISR and have a benign disease course. Therefore, lung fibrosis should be considered an exceptional complication of DISR, while representing a well‐known complication of long‐term idiopathic sarcoidosis [51].

Our study is the largest series on paradoxical sarcoidosis in IBD to date, with the description of nine new cases bringing the total number of patients to 26. The study has several limitations. First, the retrospective design may lead to several biases on the reported data. Also, the follow‐up period for new cases differs from that of cases included in the literature review, and there was an important heterogeneity in diagnostic work‐up. Finally, the sample size is relatively small, mainly given the rarity of the condition. Reporting bias may have affected the study, possibly resulting in the overlooking or nonreporting of several asymptomatic cases. On the other hand, our paper highlights the heterogeneity of DISR manifestations, the diagnostic challenges, and the possible overlap with IBD pathogenesis and manifestations. Compared to patients previously described in the literature, our report provides valuable insights into managing this rare complication and adds important information on its management. The longer follow‐up period (a median of 45 months after sarcoidosis diagnosis) with overall positive outcomes suggests a benign course of DISR. Moreover, the more recent timeframe of our report aligns better with the current therapeutic landscape, which includes many alternatives to anti‐TNF therapy. This reinforces the idea that the optimal management of DISR involves permanently discontinuing anti‐TNF therapy and introducing specific treatments for moderate‐to‐severe cases of sarcoidosis. Further worldwide prospective studies are needed to gather more information on DISR and confirm our findings.

**Nomenclature**
anti‐TNF: anti‐tumor necrosis factor‐alpha
BAL: bronchoalveolar lavage
CD: Crohn′s disease
DISR: drug‐induced sarcoidosis reaction
EIM: extraintestinal manifestation
IBD: inflammatory bowel disease
UC: ulcerative colitis

## Author Contributions

B.P., C.Z., A.A., A.M., B.Pe., B.A., S.P., N.S., and S.E. collected clinical data. B.P. and F.C. conducted the literature review and analyzed the clinical data. B.P., F.C., C.F., and R.M. wrote the draft of the paper. All the authors reviewed the manuscript before submission.

## Funding

No funding was received for this manuscript. Open access publishing facilitated by Universita degli Studi di Padova, as part of the Wiley ‐ CRUI‐CARE agreement.

## Ethics Statement

The authors have nothing to report.

## Consent

Informed consent for retrospective data collection was obtained from all patients at each center.

## Conflicts of Interest

A.A. reports serving as an advisory board from Takeda, Galapagos, Pfizer, and AbbVie. A.B. reports receiving lecture and consultancy fees from AbbVie, Aengus, Boston Scientific, Bristol‐Myers Squibb, Dr. Falk Pharma, Genericon, Gilead, Janssen, Lilly, MSD, Olympus, Pfizer, PSI, Sandoz, Takeda, and Vifor. P.S. reports receiving consulting fees from Takeda, AbbVie, Merck‐MSD, Biocodex, Janssen, Amgen, Astellas, and Pfizer and grants from Biocodex and Janssen. C.F. reports serving on the advisory boards for AbbVie, Janssen, and MSD.

## Supporting information


**Supporting Information** Additional supporting information can be found online in the Supporting Information section. Table S1: Additional characteristics of the patients, including Montreal classification [31] and extraintestinal manifestations. Table S2: Literature review of cases of anti‐TNF drug‐induced sarcoidosis in IBD. Table S3: Commonalities and differences among IBD and sarcoidosis.

## Data Availability

The data that support the findings of this study are available from the corresponding author upon reasonable request.
