# Supplementary material for: Anti‐TNF Drug‐Induced Sarcoidosis in Inflammatory Bowel Diseases: Multicentric Case Series and Literature Review
Source: Biomed Res Int. 2026 May 22;2026:7554982. doi: 10.1155/bmri/7554982 (PMC13195438; doi:10.1155/bmri/7554982)
Supplement: Supplementary file 1 — Supporting Information Additional supporting information can be found online in the Supporting Information section. Table S1: Additional characteristics of the patients, including Montreal classification [31] and extraintestinal manifestations. Table S2: Literature review of cases of anti‐TNF drug‐induced sarcoidosis in IBD. Table S3: Commonalities and differences among IBD and sarcoidosis. [file BMRI-2026-7554982-s001.docx]

**SUPPLEMENTARY MATERIAL:**

**Supplementary table 1.** Additional characteristics of the patients, including Montreal classification (31) and extrainstestinal manifestations.

| **Variable** | **CD  (N=6)** | **UC (N=2)** | **IBD-U (N=1)** |
| --- | --- | --- | --- |
| Age at IBD diagnosis (y) | 22 (range 11-37) | 17 and 56 | 20 |
| Age at sarcoidosis (y) | 42 (range 31-56) | 26 and 57 | 25 |
| Sex (F/M) | 0 (0%)/6 (100%) | 1 (50.0%)/1 (50.0%) | 0 (0%)/1(100%) |
| Age at diagnosis |  |  |  |
| A1 <17 y | 1 (16.7%) | NA | NA |
| A2 17-40 y | 4 (66.7%) | NA | NA |
| A3 >40 y | 1 (16.7%) | NA | NA |
| Location |  |  |  |
| L1-ileal involvement | 1(16.7%) | NA | NA |
| L2-colonic involvement | 1(16.7%) | NA | NA |
| L3-ileocolonic involvement | 4(66.7%) | NA | NA |
| L4-gastric involvement | 4 (66.7%) |  |  |
| Behaviour |  |  |  |
| B1-inflammatory | 1 (16.7%) | NA | NA |
| B2-stricturing | 2 (33.3%) | NA | NA |
| B3-penetrative disase | 3 (50.0%) | NA | NA |
| p-perianal_disease | 3(50.0%) | NA | NA |
| Extension |  |  |  |
| E1-proctitis | NA | 0 (0%) | 0 (0%) |
| E2-left side colitis | NA | 1(50.0%) | 0 (0%) |
| E3-pancolic involvement | NA | 1 (50.0%) | 1(100%) |
| Extraintestinal manifestations | 3 (50.0%)  Articular (n=2)*,  Skin (n=2)* | 2 (100%)  Articular (n=1),  Hepatic (n=1) | 1 (100%),  Articular (n=1) |

*One patient presented both complications. Abbreviations: y=years; F=female; M=male; CD=Crohn’s disease; UC=ulcerative colitis; IBD-U=unclassified inflammatory bowel disease.

**Supplementary Table 2. Literature review of cases of anti-TNF induced sarcoidosis in IBDs.**

| **NR** | **IBD** | **Sex** | **Anti-TNF/ age (Y)** | **Time to sarcoidosis (Y)** | **SS** | **Organ involvement** | **Histology** | **anti-TNF stop** | **Therapy** | **Reference** |
| --- | --- | --- | --- | --- | --- | --- | --- | --- | --- | --- |
| 1 | CD | M | Ada/30 | 5 | Yes | Lung/Skin/Uveitis | Yes | Yes | Steroids | Decock et al. 2017(13) |
| 2 | CD | M | Ada/21 | 1.5 | No | Lung/Skin/Heart | Yes | Yes | HCQ (+ topical Steroid); Steroids | Decock et al. 2017(13) |
| 3 | CD | F | Ifx/44 | 5 | No | Neuro (definitive) | Yes | Yes | Steroids | Simonetto et al. 2015 (16) |
| 4 | CD | M | Ada/37 | 2 | No | Lung/Skin (Loefgren-like) | Yes | Yes | Steroids | Mc Donnell et al. 2014 (23) |
| 5 | CD | F | Ada/25 | 1.5 | No | Lung/Spleen | Yes | No | Steroids | Kotze et al. 2013 (24) |
| 6 | CD | M | Ifx/35 | 0.58 | Yes | Lung/Skin | Yes | No | No | Takahashi et al. 2010 (25) |
| 7 | CD | F | Ada/49 | 10 | No | Oral | Yes | No | Steroid (topical) | Simonato 2023 (26) |
| 8 | CD | F | Ifx/35 | 1 | Yes | Lung/Liver/Spleen | Yes | Yes | Steroid | Chebli et al. 2024 (27) |
| 9 | CD | F | Ifx/37 | 15 | Yes | Lung/Liver | Yes | Yes | No | Kashima et al. 2021 (28) |
| 10 | CD | M | Ifx/18 | 2 | Yes | Lung | Yes | Yes | No | Okoshi et al. 2020 (29) |
| 11 | UC | F | Ifx/66 | 2.3 | No | Skin | Yes | No | Steroid | Fok et al. 2012 (30) |
| 12 | UC | M | Ifx/30 | 3.8 | Yes | Lung/ Liver/Sub | Yes | Yes | Steroid | Gîlcă et al. 2017 (17) |
| 13 | UC | M | Ada/42 | 1 | Yes | Lung/Kidney/Gut | Yes | Yes | Steroid | Villemaire et al. 2014 (18) |
| 14 | CD | M | Ifx/28 | 0.67 | No | Lung | Yes | Yes (only 5 months) | No | Kim et al. 2017 (19) |
| 15 | CD | M | Ifx/34 | 10.67 | Yes | Lung/Skin* | Yes | No | No | Numakura et al. 2016 (20) |
| 16 | CD | F | Ifx/57 | 6 | Yes | Lung | Yes | Yes | Steroids | Fuentes-Valenzuela et al. 2021 (21) |
| 17 | UC | F | Ada/42 | 1 | Yes | Lung/Skin | Yes | No | Steroid | Arenas et al. 2025 (22) |

Abbreviations: M=male; F=female; ibd=inflammatory bowel disease; CD=Crohn’s disease; UC=Ulcerative colitis; IBD-U=undefined IBD; Montreal=classification according to Montreal for UC and/or CD(31). EIM=extraintesinal manifestation; A=articular; S=skin; H=hepatic; IFX=infliximab; ADA=adalimumab; dx=diagnosis of IBD; y=years; SS=systemic symptoms for sarcoidosis; Neuro=neurological involvement; Lung=lung involvement; lLN=infradiaphragmaetic lymph nodes; BAL=bronchoalveolar lavage. USTE=Ustekinumab; UPA=Upadacitinib; VEDO=vedolizumab. *Concomitant leukocytoclastic vasculitis not considered as part of DISR.

**Supplementary table 3.** Commonalities and differences among IBD and Sarcoidosis.

| Disease | **IBD** | **Sarcoidosis** |
| --- | --- | --- |
| Granuloma | CD: yes UC: no | Yes |
| Primary involvement* | Gastrointestinal segment involvement:  Any segment in CD  Colic involvement in UC | Intrathoracic involvement - Hilar-mediastinal lymph nodes - Lung parenchyma  - Lung Fibrosis |
|  | **Extraintestinal manifestations** | **Extra-thoracic involvement** |
| Joints | Arthalgia  Peripheral arthritis | |
|  | Axial arthritis Enteropathic arthritis |  |
| Skin | Erythema nodosum | |
|  | Pyoderma gangrenosus Metastatic CD Other | Lupus pernio Subcutaneous nodules Scar sarcoidosis Other |
| Eye | Uveitis  (Epi)Scleritis | |
|  |  | Conjuntivitis  Lacrimal gland involvement |
| Liver | Granolomatous hepatitis | |
|  | Primary sclerosing cholangitis  Autoimmune hepatitis |  |
| Lymph node | Not typical | Enlargement:  - Upper diaphragmatic - Lower diaphragmatic |
| Kidney | Nephrolitiasis | Hypercalciuria/hypercalcemia granulomatous interstitial nephritis |
| Bone and muscle | Not typical | More frequently asymptomatic |
| Heart | Not typical | Asymptomatic  Arrhytmias Heart failure |
| Central nervous system | Not typical | Cranial nerves involvement meningitis brain parenchyma spinal cord involvement Hypophysis-hypothalamus |
| Pancreas | Pancreatitits | Rarely described |
| Spleen | Not typical | Splenomegaly Granuloma |
| Venous thrombosis | Especially in active disease | Not typical |

The manifestations that are reported as justified text are shared by both diseases. *Rarely, IBD and sarcoidosis may present with lung and gastrointestinal involvement, respectively. Abbreviations: IBD=inflammatory bowel disease; CD=Crohn’s disease; UC=Ulcerative colitis.
